# Psychisch kranke Bürgerinnen und Bürger als Opfer von Eingriffen in körperliche Unversehrtheit und Vernichtung in der NS-Zeit am Beispiel einer deutschen Stadt

**DOI:** 10.1007/s00115-024-01745-y

**Published:** 2024-09-26

**Authors:** Beatrice Rose, Jörg-Stefan Schulz, Eckhard Sundermann, Paraskevi Mavrogiorgou, Georg Juckel

**Affiliations:** 1 Klinik für Psychiatrie, Psychotherapie und Präventivmedizin, LWL-Universitätsklinikum, Ruhr-Universität Bochum, Alexandrinenstr. 1, 44791 Bochum, Deutschland; 2 Institut für Medizinische Ethik und Geschichte der Medizin, Ruhr-Universität Bochum, Bochum, Deutschland

**Keywords:** Euthanasie, T4-Aktion, Zwangssterilisation, Psychiatrische Individualgeschichte, Euthanasia, T4 Action, Forced sterilization, Individual psychiatric history

## Abstract

**Hintergrund:**

Auch 90 Jahre nach nationalsozialistischer Machtergreifung und Beginn des dunkelsten Kapitels deutscher Psychiatriegeschichte bleibt die Auseinandersetzung mit der medizinhistorischen Vergangenheit als kontinuierliche ärztliche Verantwortung von großer Relevanz. Die Eingriffe in das Leben psychisch kranker Menschen durch das nationalsozialistische Regime sind zwar ein fest etablierter Bestandteil der medizinhistorischen Forschung; über die genauen Vorgänge in einer konkreten Region mit ihren betroffenen Bürgerinnen und Bürgern ist jedoch bisher wenig bekannt.

**Ziel der Untersuchung:**

Ziel dieser Untersuchung war es daher, Verlegungsrouten der „Euthanasie“-Transporte und Sterbeorte der Bochumer Opfer sowie Patientencharakteristika der Betroffenen zu identifizieren.

**Material und Methoden:**

Zum untersuchten Zeitpunkt erfolgte die stationäre Versorgung psychisch kranker Patientinnen und Patienten aus dem Raum Westfalen in sieben Provinzialanstalten. In Bochum gab es damals keine psychiatrische Klinik, sodass die Patientinnen und Patienten auf diese Anstalten verteilt wurden. Die Basis dieser Untersuchung bildeten die Verlegungslisten der westfälischen Provinzialanstalten in die Euthanasie-Anstalten.

**Ergebnisse und Diskussion:**

Es wurden insgesamt 366 Bochumer Bürgerinnen und Bürger identifiziert, die von „Euthanasie“-Verlegungen betroffen waren. Die Transportlisten wurden durch Aufnahme- und Abgangsbücher, Sterbelisten sowie Patientenakten verifiziert. Mithilfe der Erbgesundheitsakten des Stadtarchivs konnten die verlegten Bochumer Patienten daraufhin untersucht werden, ob sie Opfer einer Zwangssterilisation wurden. Das Untersuchungsprojekt war durch die DGPPN-Wanderausstellung zur T4-Aktion angestoßen worden und ist Teil des Bochumer Stolpersteinprojekts.

## Abstract (en)

**Background:**

Even 90 years after the National Socialist seizure of power and the beginning of the darkest chapter of German psychiatric history, examination of the medical historical past retains great relevance as an ongoing medical responsibility. The interventions in the lives of mentally ill people by the National Socialist regime are a firmly established part of medical historical research; however, little is known about how specific regions proceeded with affected citizens.

**Objective:**

The aim of this study was therefore to identify transfer routes of the “euthanasia” transports and places of death of the victims from Bochum as well as their patient characteristics.

**Materials and methods:**

During the timeframe studied, inpatient care for mentally ill patients from the Westphalia region was provided in seven provincial institutions. There was no psychiatric clinic in Bochum at the time, so patients were distributed among these institutions. The investigation was based on the transfer lists of the Westphalian provincial institutions to the killing sites.

**Results and conclusion:**

A total of 366 Bochum citizens affected by “euthanasia” transfers were identified. The transport lists were verified by admission and departure books, death lists, and patient files. The hereditary health files of the city archive were used to examine the relocated Bochum patients to determine whether they were victims of forced sterilization.

## Einleitung

Die nationalsozialistische Ideologie der Ausgrenzung sogenannter „rassenfremder“ und „lebensunwerter“ Bevölkerungsgruppen stützte sich auf Konzepte der Eugenik und des Sozialdarwinismus, welche schon zum Ende des 19. Jahrhunderts international verbreitet waren [8, 22, 33]. In Deutschland kritisierten insbesondere Ärzte das Fehlen einer „natürlichen“ Auslese und warnten vor der „Degeneration“ der Bevölkerung [12]. Ernst Haeckel propagierte den Sozialdarwinismus („Selektionstheorie“; [14, 39]). Ausgehend von seiner Hypothese, dass die menschliche Seele ebenso wie Haut- und Haarfarbe vererbt werde, sprach sich Haeckel für die negative Eugenik aus, um der „Entartung“ der Bevölkerung entgegenzuwirken [14, 29]. In Deutschland waren um das Jahr 1890 die Ärzte Alfred Ploetz und Wilhelm Schallmayer Begründer von Kernthesen der Rassenhygiene [29, 31]. Ploetz stellte in seinem Werk „Die Tüchtigkeit unserer Rasse und der Schutz der Schwachen“ 1895 ein Gedankenkonstrukt einer „rassenhygienischen Utopie“ vor und veröffentlichte ab 1904 die Zeitschrift *Archiv für Rassen- und Gesellschafts-Biologie*, ein zentrales Medium für die Veröffentlichungen eugenischer Theorien [12, 26]. Der Psychiater Alfred Hoche und der Jurist Karl Binding sprachen sich 1920 in ihrem Werk „Die Freigabe der Vernichtung lebensunwerten Lebens“ für die Ermordung „unheilbar Kranker“ und „unheilbar Blödsinniger“ aus und plädierten für die Minimierung von sogenannten „Ballastexistenzen“, die die „Leistungsfähigkeit“ der Gesellschaft durch die Beanspruchung finanzieller Mittel und kontinuierlicher Pflege beeinträchtigten [2]. Im Folgejahr (1921) veröffentlichten der Anthropologe Erwin Bauer, der „Züchtungsforscher“ Eugen Fischer und der Rassenhygieniker Fritz Lenz das Werk „Grundriß der menschlichen Erblichkeitslehre und Rassenhygiene“, das als wissenschaftliche Grundlage für das 1934 in Kraft gesetzte nationalsozialistische Sterilisationsgesetz diente, aufgrund dessen bis Kriegsende Hunderttausende Menschen sterilisiert wurden [4, 12, 39]. Somit kam es zur Instrumentalisierung und Funktionalisierung der damals wissenschaftlich weithin anerkannten Rassenhygiene, um nationalsozialistische Ideologie zu etablieren; eine „intellektuelle“ und „utilitaristische“ Wechselbeziehung zwischen der Rassenhygiene und dem Nationalsozialismus schuf somit Seriosität und politische Handlungsfähigkeit [39].

Drängen von Medizinern nach einem eugenischen Sterilisationsgesetz ließ die Ärztekammer für Westfalen am 25.03.1933 schließlich fünf Leitsätze für dessen Umsetzung verabschieden, darunter die folgenreiche Forderung nach Zwang [38]. Das „Gesetz zur Verhütung erbkranken Nachwuchses“ wurde in der Kabinettssitzung der neuen nationalsozialistischen Regierung vom 14.07.1933 verabschiedet, am 25.07.1933 veröffentlicht und am 01.01.1934 in Kraft gesetzt. Die „Optimierung“ der Bevölkerung durch Steigerung des Nachwuchses der „wertvolleren“ Bevölkerungsschichten über jenen der „wertlosen“ war übergeordnetes Ziel des Sterilisationsgesetzes [4]. Als erbkrank kategorisiert wurden: „angeborener Schwachsinn“, „Schizophrenie“, „zirkuläres Irresein“, „erbliche Fallsucht“, „erbliche Blindheit und Taubheit“, „erblicher Veitstanz“, „schwere erbliche körperliche Behinderung“ und „Alkoholismus“ [27, 38]. Am 05.12.1933 veröffentlichten das Reichsjustizministerium und Reichsinnenministerium eine Zusatzverordnung, in der den Betroffenen ein Aufschub der Sterilisation bei freiwilliger Einweisung in eine psychiatrische Anstalt gewährt wurde [27]. Ärzte wurden nun angehalten, „erbkranke“ Menschen zu erfassen und Anträge auf Zwangssterilisierung zu stellen, über die nun neu geschaffene Erbgesundheitsgerichte, die aus zwei Ärzten und einem Juristen bestanden, entschieden. Dieser Sterilisationspraxis fielen zwischen 1934 bis 1945 etwa 400.000 Menschen beiderlei Geschlechts zum Opfer, wobei es bei den weiblichen Opfern nicht selten postoperativ zu Todesfällen kam [4, 19]. In der Hälfte der Fälle wurde die Diagnose „angeborener Schwachsinn“ dokumentiert, einem Viertel der Sterilisationen lag die Diagnose „Schizophrenie“ zugrunde, in 14 % lautete sie „Epilepsie“ [12]. Im Oktober 1935 wurde psychisch Kranken schließlich die Eheschließung durch die Verabschiedung des „Ehegesundheitsgesetzes“ untersagt [27].

Hitlers Anweisung im Oktober 1939, die er auf Kriegsbeginn zurückdatierte, leitete die nächste Eskalationsstufe nationalsozialistischer Terrorherrschaft ein: „Reichsleiter Bouhler und Dr. med. Brandt sind unter Verantwortung beauftragt, die Befugnisse namentlich zu bestimmender Ärzte so zu erweitern, dass nach menschlichem Ermessen unheilbar Kranke bei kritischster Beurteilung ihres Krankheitszustandes der Gnadentod gewährt werden kann“ [12, 23]. In der sogenannten „Aktion T4“ wurde die Tötungslegitimation zum „Gnadentod“ psychisch kranker Menschen umgesetzt. Euphemistisch als „Euthanasie“ bedeutete dieser letztlich das Todesurteil von mehr als 70.000 psychisch kranken Menschen [1, 8, 12, 25, 34, 35]. In kürzester Zeit wurden nun alle psychiatrischen Einrichtungen des Reiches durch die Reichsarbeitsgemeinschaft der Heil- und Pflegeanstalten des „T4-Apparates“ aufgefordert, Meldebögen anzufertigen [29]: Im „Meldebogen 1“ wurden Patienteninformationen wie Arbeitsleistung, Diagnose und Aufenthaltsdauer eingefordert [16, 36, 38]. Der „Meldebogen 2“ diente der Erfassung von baulichen Gegebenheiten, Bettenkapazität, derzeitiger Belegung und Personalschlüssel. Im Bereich Westfalen wurde dem Provinzialverband die Aufsichtspflicht des Meldeprozesses zuteil [38].

Wie westfälische Regionalforschungen fanden, zeigte die Modalität der Meldung psychiatrischer Patientinnen und Patienten im westfälischen Raum regionale Unterschiede: Die Meldequote korrelierte mit der Verlegungsquote und somit auch mit der Anzahl der „potenziellen Euthanasieopfer“ [38]. Die Heilanstalten mit den höchsten Meldequoten von 92 bis 99 % waren Lengerich, Warstein, Eickelborn und Marsberg [38]. Strategisch ausgeklügelte Verlegungsrouten führten 2890 westfälische psychiatrische Patienten im Zeitraum vom 24. Juni bis zum 26. August 1941 in die zu diesem Zweck umstrukturierten „Zwischenanstalten“ Herborn, Eichberg, Scheuern, Kalmenhof, Weilmünster und viele schließlich ins hessische Hadamar [38]. Von diesen wurden 46 % in der dort im Keller befindlichen Gaskammer der zur „Tötungsanstalt“ umgebauten Heil- und Pflegeanstalt ermordet [38]. Katastrophale Versorgungsumstände und das inoffizielle Fortführen der „Euthanasie“ durch Ärzte und Pflegepersonal ließen die Sterblichkeit in den Verlegungskliniken jedoch auf einen Höchstwert von 75 % anwachsen [18, 37, 38].

Die Verschärfung der Luftkriegslage 1943 führte schließlich zur sogenannten „Aktion Brandt“; psychiatrische Anstalten wurden geräumt, um medizinische Versorgungskapazitäten der im Krieg zerstörten Krankenhäuser zu sichern. Die Westfälischen Provinzialanstalten, kategorisiert nach ihrem Gefährdungspotenzial für Luftkriegsangriffe, wurden angewiesen, Kapazitäten zu schaffen, indem psychisch kranke Patientinnen und Patienten verlegt wurden. Von der Reichsarbeitsgemeinschaft Heil- und Pflegeanstalten koordiniert, wurden in diesen Jahren insgesamt 2846 westfälische Psychiatriepatienten in süd- und ostdeutsche Gebiete verlegt [9]. Da Bochum keine eigene psychiatrische Klinik aufwies, wurden die psychiatrisch kranken Bochumer Bürger traditionell in den Anstalten des Westfälischen Provinzialverbands (Nachfolgeorganisation Landschaftsverband Westfalen-Lippe), die sich vor allem östlich des Ruhrgebiets, wie in Eickelborn, Warstein, Dortmund, Marsberg usw. befanden, versorgt.

## Methode

### „Euthanasie“

Der Sammelbestand 820 des LWL-Archivamts Münster diente als Grundlage der Untersuchung. Die Listen des Bestands 840-22/1‑7 sind Kopien aus den Überlieferungen der Heil- und Pflegeanstalten der Region. Hierbei handelt es sich um die Dokumentation der Transporte psychisch kranker Patientinnen und Patienten, die auf Veranlassung der Militärregierung nach 1945 erstellt worden war.

Mithilfe der Listen über die Verlegungen der psychiatrischen Patientinnen und Patienten in der Region Westfalen konnten Bochumer Bürgerinnen und Bürger identifiziert werden, indem die Verlegungslisten auf das Hauptmerkmal Wohnort Bochum durchgeschaut wurden. Der Geburtsort wurde nur als Zusatzinformation gewertet. Dies wurde versucht zu verifizieren, indem die Aufnahmelisten der Westfälischen Provinzialanstalten für das Hauptmerkmal „Zuverlegung aus Bochum“ auf Anordnung des zuständigen Arztes im Gesundheitsamt Bochum überprüft wurden. Die Gesamtzahl der so definierten und gefundenen Bochumer Patientinnen und Patienten, die der Euthanasie anheimfielen, betrug *n* = 366.

In den Verlegungslisten wurden auch das Datum der Verlegung und der Ort der Zielklinik sowie Entlassungen dokumentiert. Ferner wurden lückenhaft Angaben über das Versterben der Patientinnen und Patienten, wie Sterbedatum, Todesursache und Sterbeort, festgehalten. Diese Informationen wurden in Zusammenschau mit biografischen Informationen aus den folgend dargestellten Quellen analysiert.

Die Patientenakten selbst wurden zwar mit jeder Verlegung an die Aufnahmeanstalt übermittelt, aber nur wenige Patientinnen und Patienten verblieben im Raum Westfalen. Der Aktenbestand des LWL-Archivamts Münster bewahrt daher nur wenige Patientenakten von Bochumer Bürgerinnen und Bürgern, die aus der LWL-Klinik Gütersloh und aus der LWL-Klinik Münster stammen (zwei Patientenakten aus dem Bestand 661, LWL-Klinik Gütersloh; eine Patientenakte aus dem Bestand 658, LWL-Klinik Münster).

In der detaillierten Analyse der Patientenmerkmale wurden nur die Bochumer Patientinnen und Patienten aufgrund der Aktenlage aufgenommen, die in westfälische und hessische Kliniken verlegt wurden. In der Regel wurden diese Patienten zunächst in sogenannte „Zwischenanstalten“ verlegt. Zu diesen zählen im westfälischen und hessischen Raum: Weilmünster, Eichberg, Kalmenhof, Scheuern und Herborn.

In der Datenerhebung wurden die Aufnahmebücher der Zwischenanstalt Weilmünster auf Bochumer Patientinnen und Patienten untersucht (B19, 13 LWV-Archiv: Zugangsliste der Kranken 1933–1941, B19, 14 LWV-Archiv: Aufnahme- und Abgangsbücher Männer und Frauen 1937–1941, B19, 16 LWV-Archiv: Aufnahme- und Abgangsbücher Männer und Frauen 1941–1961). Den Aufnahmebüchern konnten Informationen über den Zeitpunkt der Aufnahme und der Verlegung aus den „Zwischenanstalten“ entnommen und so Informationen aus den Verlegungslisten verifiziert werden. Ferner finden sich lückenhaft Angaben über die Zielanstalt nach Verlegung und Sterbeorte der Patientinnen und Patienten.

Die Bestände des LWV-Archivs Kassel umfassen auch Informationen über die Provinzialanstalt Eichberg. Der Aktenbestand B10 85 beinhaltet die Auflistung von in Eichberg verstorbenen Patientinnen und Patienten. Die Eichberger Sterbeliste mit der Signatur LWV-Archiv, B 10 Nr. 85 und das Aufnahmebuch der Provinzialanstalt Eichberg mit der Signatur LWV-Archiv, B 10 Nr. 120 wurden auf Bochumer Patientinnen und Patienten überprüft. Weiterhin wurden die Aufnahmebücher der Provinzialanstalt Scheuern auf Bochumer Patientinnen und Patienten untersucht (BA-Signatur 25240 Heime Scheuern, HKV [Scheuern]). Auch die Aufnahmebücher der Provinzialanstalt Herborn wurden auf Aufnahme Bochumer Patientinnen und Patienten hin untersucht (Bestand 461/32061, Band 17 Liste Herborn [Wiesbaden]).

Die Patientenakten von Bochumer Bürgerinnen und Bürgern, die nach Hadamar verlegt wurden, werden im Archiv der Gedenkstätte Hadamar, einer Außenstelle des Archivs des Landeswohlfahrtsverbands Hessen LWV in Kassel, archiviert. Der Bestand „K 12 Hadamar“ des LWV-Archivs umfasst die Patientenakten der Menschen, die der dezentralisierten „Euthanasie“-Phase in der ehemaligen „Tötungsanstalt“ Hadamar zum Opfer fielen. Die Opferdatenbank ist in zwei Rubriken gegliedert: Euthanasie-Phase 1 bis 1941 und Euthanasie-Phase 2 1942–1945. Die Patientenakten der ersten „Euthanasie“-Periode sind in der Gedenkstätte Hadamar nicht archiviert (teilweise überliefert im Bundesarchiv Bestand R179, wie Untersuchungen von Hohendorf zeigten [16]). Insgesamt umfasst dieser Bestand circa 4000–5000 Akten aus den Jahren 1942–1945. Die Untersuchung des Bestands „K 12“ lieferte insgesamt 33 Patientenakten von Bochumer Patientinnen und Patienten.

### Zwangssterilisation

Die Sterilisationsprozesse wurden in den Erbgesundheitsakten dokumentiert und vom Gesundheitsamt verwaltet. Der Bestand des Bochumer Stadtarchivs umfasst circa 3500 Erbgesundheitsakten des Gesundheitsamts in Bochum. Die 366 Bochumer Opfer der „Euthanasie“ wurden schließlich mithilfe des Bestands „BO-53“ des Bochumer Stadtarchivs darauf untersucht, ob sie zwangssterilisiert wurden.

Dieser Bestand umfasst weiterführende biografische Daten (Geburtsdatum, Geburtsort, Eheschließung), die in der Gesamtanalyse ergänzt wurden. Teilweise konnten zusätzliche Informationen über Krankheitsbeginn und -ausprägung sowie die Familienanamnese gewonnen werden. Die Zeitachse des Sterilisationsprozesses der psychisch kranken Bochumer Bürgerinnen und Bürger konnte von der Antragstellung auf Unfruchtbarmachung mit begründender psychischer Erkrankung bis zum Zeitpunkt der Durchführung rekonstruiert werden. Neben Sterilisationsanträgen finden sich in diesem Bestand auch Stammbäume, sogenannte „Sippentafeln“. Die Auswertung der Stammbäume ermöglichte weiterführende Einblicke in die Familienanamnese der Patientinnen und Patienten.

Die Zusammenschau der Informationen aus den genannten Quellen erlaubt eine Rekonstruktion der Lebenswege von psychisch erkrankten Bochumer Bürgerinnen und Bürgern im Nationalsozialismus, die Opfer von Eingriffen in körperliche Unversehrtheit und Vernichtung wurden. Es wurden folgende Merkmale, die in den Akten entsprechend damals notiert worden waren, quantitativ untersucht: Alter, Geschlecht, Diagnose, Verlegungsroute der Opfer, Todesursache, Konfession, Sterilisation.

## Ergebnisse

### Bochumer Opfer der „Euthanasie“-Verlegungen

Am 21. September 1940, bereits vor Durchführung der „Aktion T4“ in Westfalen, wurde ein Bochumer Patient jüdischen Glaubens aus der Provinzialanstalt Dortmund-Aplerbeck nach Wunstorf und indiziell in die „Tötungsanstalt“ Brandenburg weiterverlegt [11, 38]. Wunstorf fungierte dabei als Sammelstelle jüdischer Patientinnen und Patienten zum Weitertransport in die Tötungsanstalt.

#### Bochumer Opfer in der „Aktion T4“

In der „Aktion T4“ wurden insgesamt 149 Bochumer Patienten aus westfälischen Provinzialanstalten verlegt und 49 unmittelbar nach Verlegung in die hessischen „Zwischenanstalten“ nach Hadamar weiterverlegt. Der Großteil der verlegten Bochumer Patienten stammte aus den Provinzialanstalten Warstein (64) und Marsberg (31). Während die anderen westfälischen Anstalten hinsichtlich der Anzahl verlegter Patienten im Mittelfeld anzuordnen sind (10–15), nimmt die Provinzialanstalt Münster mit nur fünf verlegten Bochumer Bürgern eine Sonderstellung ein. Der Großteil der Bochumer Opfer wurde in die Zwischenanstalten Weilmünster (47) und Eichberg (41) verlegt. Die „Zwischenanstalten“ Scheuern (21), Kalmenhof (21) und Herborn (20) weisen einen deutlich geringeren Patientenanteil auf. Von den in der „Aktion T4“ nach Hadamar weiterverlegten Bochumer Patientinnen und Patienten stammte ein Großteil ursprünglich aus den Provinzialanstalten Warstein (25) und Marsberg (12). Bemerkenswert ist, dass im Jahr 1941 aus den Provinzialanstalten Eickelborn und Münster keine Bochumer Bürgerinnen und Bürger nach Hadamar weiterverlegt worden sind. Diese verblieben zunächst in den „Zwischenanstalten“ Scheuern und Eichberg, in denen sie nach offizieller Beendigung der „Aktion T4“ verblieben waren.

#### Bochumer Opfer in der „Aktion Brandt“

In der „Aktion Brandt“ wurde der Großteil der Bochumer Patienten aus der Provinzialanstalt Eickelborn nach Süddeutschland verlegt (105). Die Provinzialanstalten Marsberg (31), Warstein (31), Dortmund-Aplerbeck (24) und Gütersloh (22) lassen sich anhand ihrer Verlegungszahlen im Mittelfeld einordnen. Die Provinzialanstalt Münster (7) hebt sich erneut deutlich hiervon ab. Das Ziel der Verlegungen des Jahres 1943 waren für die meisten Bochumer Patientinnen und Patienten die süddeutschen Heil- und Pflegeanstalten (129) Kaufbeuren, Regensburg und Erlangen. Eine deutlich geringere Zahl wurde in westfälische Anstalten (25) und ostdeutsche Gebiete (17) verlegt. Aus den Verlegungen der „Aktion Brandt“ wurden zwölf Bochumer Bürger nach Hadamar verlegt. Ein Großteil wurde der heute auf polnischen Boden befindlichen „Tötungsanstalt“ Meseritz (26) zugeführt.

#### Ereignisse nach den Verlegungen

Die Betrachtung der Sterbeorte zeigt, dass 1941 der Großteil der Bochumer Verlegungspatienten in Hadamar starb (44).

Im Folgejahr kam es vorwiegend in den „Zwischenanstalten“ Eichberg (16), Weilmünster (9), Scheuern (4) und Kalmenhof (3), in denen sie nach der Beendigung der „Aktion T4“ und dem Aussetzen der Transporte in die „Tötungsanstalt“ Hadamar verblieben sind, zu Todesopfern. Ab 1943 und mit der „Aktion Brandt“ zeigte sich eine breite Streuung der Sterbeorte auch in süd- und ostdeutsche Gebiete. Die Anzahl der Bochumer Todesopfer nahm rapide zu (77). Ein Großteil von ihnen verstarb in den „Tötungsanstalten“ Hadamar (24) und Meseritz (22) und viele fanden den Tod in den „Zwischenanstalten“ Weilmünster (9) und Eichberg (5). Die Verlegungen im Rahmen der „Aktion Brandt“ in periphere Gebiete führten auch zu Bochumer Todesopfern in Erlangen (5), Bernburg (4), Kaufbeuren (3) und Eglfing-Haar (2). In den westfälischen Provinzialanstalten Aplerbeck (1) und Lengerich (1) starben nur wenige Personen des betrachteten Patientenkollektivs.

**Abb. 1:**
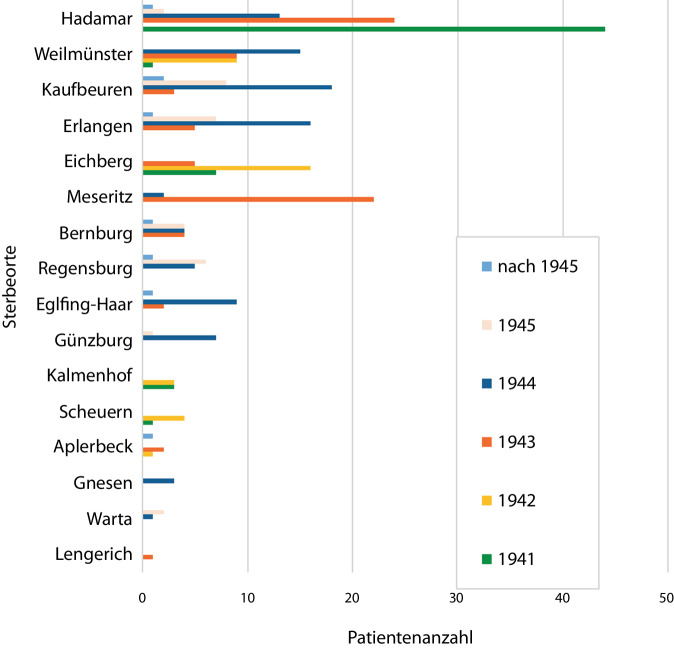
Sterbeorte Bochumer Opfer 1941–1945

Im Folgejahr 1944 kam es zu einem Höchstwert Bochumer Todesopfer (93). Ab 1945 zeigt sich ein deutlicher Rückgang der Anzahl Bochumer Todesopfer (30); der Großteil starb in den süddeutschen Anstalten Kaufbeuren (8), Erlangen (7) und Regensburg (6). Abb. 1 stellt die Sterbeorte Bochumer Opfer in den Jahren 1941 bis 1945 dar.

### Patientencharakteristika

**Abb. 2:**
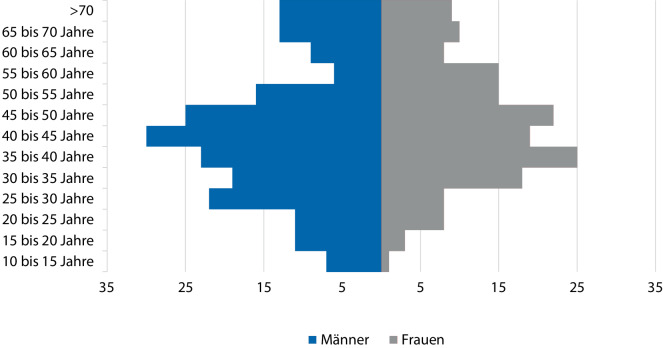
Alter (in Jahren) der Bochumer Opfer zum Verlegungszeitpunkt

Von den betroffenen 366 Bochumer Bürgerinnen und Bürgern waren 205 männlich und 161 weiblich. Durchschnittlich waren die Patienten zum Zeitpunkt der ersten Verlegung 43,9 Jahre alt (Median 42,7 Jahre), die Patientinnen 44 Jahre alt (Median 42,9 Jahre). Abb. 2 veranschaulicht die Altersverteilung der Bochumer Patientinnen und Patienten zum Verlegungszeitpunkt.

**Abb. 3:**
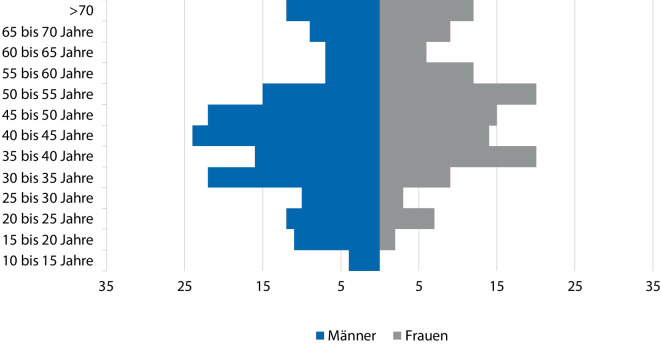
Sterbealter der Bochumer Opfer

Das Sterbealter von 171 Patienten und 129 Patientinnen ist überliefert. Durchschnittlich starben die Patienten im Alter von 45,2 Jahren (Median 44,6), die Patientinnen im Alter von 45,3 Jahren (Median 44,6). Der jüngste Patient ist im Alter von 13 Jahren und die jüngste Patientin im Alter von 17 Jahren gestorben. Der älteste Patient wurde 84 Jahre und die älteste Patientin 86 Jahre alt. Das Sterbealter der Bochumer Opfer wird in Abb. 3 visualisiert. Diagnosen von 167 Bochumer Opfern sind aktenkundig.

**Abb. 4:**
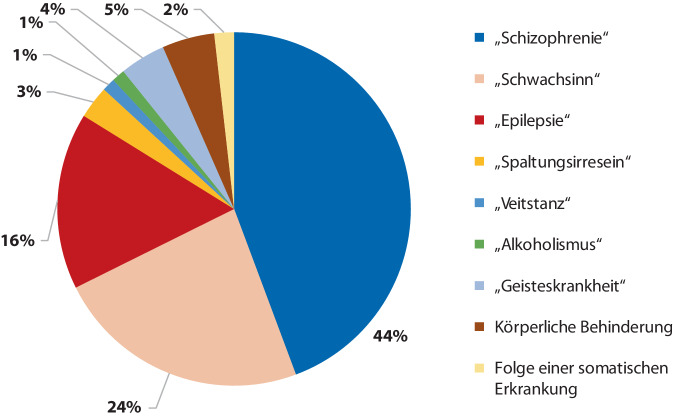
Diagnosen Bochumer Opfer

Wie aus Abb. 4 ersichtlich, wurde als häufigste zeitgenössische Diagnose „Schizophrenie“ in 44 % verzeichnet. Als zweithäufigste Diagnose wurde mit 24 % „angeborener Schwachsinn“ in den Akten angegeben. Die dritthäufigste Diagnose stellte mit 16 % „Epilepsie“ dar. Davon abzugrenzen, wurden in seltenen Fällen eine körperliche Behinderung (5 %), „Spaltungsirresein“ (3 %), „Alkoholismus“ (1 %) und „Veitstanz“ (1 %) diagnostiziert. In insgesamt 4 % fand keine Differenzierung der Diagnose statt („Geisteskrankheit“, „einfache Seelenstörung“). In 2 % der Fälle wurden die Patientinnen und Patienten als Folge einer infektiösen und vaskulären Erkrankung als „geisteskrank“ befunden.

**Abb. 5:**
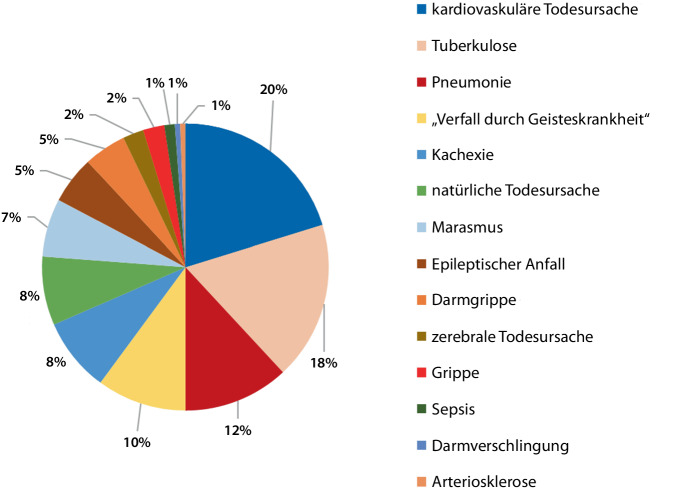
Todesursachen aus den Krankenakten oder Anstaltslisten für die Bochumer Opfer

Die Untersuchung der Todesursachen ergab, dass im Großteil der Fälle mit 20 % eine kardiovaskuläre Ursache dokumentiert wurde, gefolgt von 18 % Tuberkulose und 12 % pulmonaler Erkrankung. Als vierthäufigste Todesursache wurde in den Akten die psychische Erkrankung der Patientinnen und Patienten selbst angegeben; hierbei als häufige Formulierung „Verfall durch Geisteskrankheit“ [27]. In 8 % wurde eine Kachexie als Todesursache dokumentiert; etwa ebenso häufig wie „Marasmus“ und „natürliche Todesursache“. In 5 % wurde ein „epileptischer Anfall“ und „Arteriosklerose“ dokumentiert. Es fanden sich gehäuft Todesursachen wie „Darmgrippe“, „Darmverschlingung“, zerebrale und infektiöse Todesursachen, jedoch in deutlich geringerem Ausmaß (1–2 %). Die dokumentierten Todesursachen Bochumer Opfer sind in Abb. 5 dargestellt.

### Bochumer Patientinnen und Patienten als Opfer von Zwangssterilisation

Bei insgesamt 49 Bochumer Patientinnen und Patienten, die später im Rahmen der Euthanasie verfolgt wurden, kam es auch zur Sterilisation von Amtswegen. Dabei ist die Durchführung des Eingriffs in 40 Fällen dokumentiert. Unter den Betroffenen befanden sich 27 Männer und 13 Frauen. Die Männer waren zum Zeitpunkt der Zwangssterilisation durchschnittlich 28 und die Frauen 32 Jahre alt. Die drei häufigsten Diagnosen, aufgrund derer die Bochumer Opfer zwangssterilisiert wurden, waren an erster Stelle „Schizophrenie“ (52 %), gefolgt von „angeborenem Schwachsinn“ (30 %) und „Epilepsie“ (10 %). Seltener wurden Patienten aufgrund von „Alkoholismus“ (5 %) und „Spaltungsirresein“ (3 %) zwangssterilisiert.

**Abb. 6:**
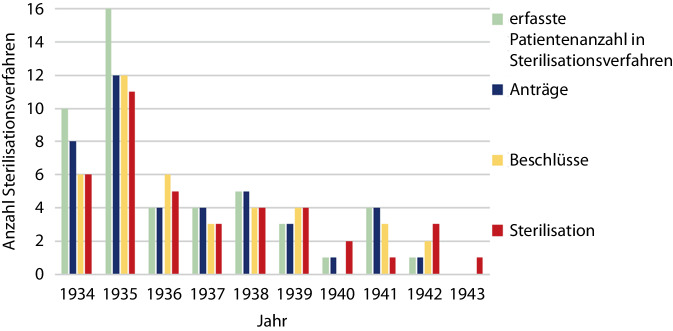
Zeitliche Entwicklung der Sterilisationsprozesse der Bochumer Opfer

Wie Abb. 6 zu entnehmen, zeigte sich eine zeitliche Dynamik in der Verteilung der Anträge, der gefassten Beschlüsse und der durchgeführten Sterilisationen in der Gruppe der verlegten Patientinnen und Patienten. Während in den ersten zwei Jahren nach Verabschiedung des Sterilisationsgesetzes etwa die Hälfte der Sterilisationsanträge gestellt wurde und mit 60 % ein Großteil der Eingriffe zeitnah durchgeführt wurde, stellt sich in den Folgejahren ein Plateau einer geringeren Anzahl von Sterilisationsanträgen ein. Auch die Zeitspanne zwischen Antragstellung und Durchführung der Zwangssterilisation zeigte sich progredient. Ab 1939 lässt sich bei verminderter Anzahl von Sterilisationsprozessen eine zeitnahe Abwicklung der angezeigten Zwangssterilisationen vernehmen. Das letzte Bochumer Opfer wurde im Jahr 1943 zwangssterilisiert.

## Diskussion

Es zeigte sich in den Verlegungslisten die frühe Deportation eines Bochumer Patienten jüdischen Glaubens aus der Provinzialanstalt Aplerbeck nach Wunstorf, also vor der Aktion „T4“. Nach nationalsozialistischer Ideologie zwei Ausgrenzungsfaktoren vereinend, wurde dieser Patient nicht anhand von „T4-Auswahlkriterien“ selektiert, sondern vielmehr aufgrund seiner Konfession, die im Nationalsozialismus grundlegend rassistisch als eine „Bedrohung für die Volksgemeinschaft“ bewertet wurde [6, 16, 24, 25]. Dieses Einzelschicksal kann als Verweis auf den Beginn des antisemitisch motivierten Genozids betrachtet werden und reiht sich in bisherige Forschungsergebnisse ein: So wertet Friedlander die frühe Deportation jüdischer psychisch kranker Menschen als „wichtiges Bindeglied zwischen Euthanasie und Endlösung“, Finzen interpretiert diese als „Generalprobe“ des Holocausts, Gruner zeigt diesbezüglich „Übereinstimmungen und Überschneidungen“ auf und Leidinger postuliert einen „inhaltlichen und organisatorischen Zusammenhang zwischen [Holocaust und Euthanasie]“ [7, 11, 12, 24].

Die Verlegungen der „Aktion T4“ zeigten zunächst einen überraschend geringen Patientenanteil Bochumer Bürgerinnen und Bürger aus den Provinzialanstalten Lengerich (15) und Eickelborn (12) im Vergleich zu Walters Regionalforschungen zum westfälischen Gesamtpatientenkollektiv [12]. Während die geografische Distanz Lengerichs zur Stadt Bochum und eine geringere Bettenkapazität (1171 im Jahr 1941) im westfälischen Vergleich einen plausiblen Erklärungsansatz für die Anzahl verlegter Bochumer Opfer trotz hoher Verlegungsquoten bieten, sind die vorliegenden Ergebnisse der Eickelborner Verlegungszahlen unerwartet [32, 38]. Die Provinzialanstalt Eickelborn lässt sich sowohl anhand ihrer Kapazität (1515 im Jahr 1941) als auch anhand der geografischen Distanz zur Stadt Bochum im Mittelfeld anordnen und ließe durch Spitzenwerte der Verlegungsquoten eine höhere Anzahl verlegter Bochumer Bürgerinnen und Bürger in der „Aktion T4“ vermuten [32, 38]. Interne Selektionskriterien im Meldeprozess, wie die „Arbeitsleistung“ und „Anstaltsaufenthaltsdauer“, könnten hierfür als mögliche Erklärungsansätze in Betracht gezogen werden [32, 38]. Ein „an Nützlichkeitserwägungen orientierter Selektionsprozess“ der „T4“-Opfer ist in der „Euthanasie“-Forschung vielfach postuliert und somit auch bei den Bochumer Opfern denkbar [6, 16, 28, 30, 34].

In den späteren Verlegungen der „Aktion Brandt“ 1943 hebt sich hingegen insbesondere die Provinzialanstalt Eickelborn mit einem Großteil verlegter Bochumer Bürger (105) bei vergleichsweise geringerer Verlegungsquote (27 %) deutlich ab [38]. Als Besonderheit für die Stadt Bochum kombinierte die Provinzialanstalt Eickelborn eine gewisse geografische Nähe und ein geringes Gefährdungspotenzial für Luftkriegsangriffe, sodass sie ein attraktives Ziel für ein Ausweichkrankenhaus der bedrohten Rhein-Ruhr-Region darstellte. Nach Räumung Eickelborns nahm die Bochumer Gynäkologie die neu geschaffenen Versorgungskapazitäten ein: Paradoxerweise übte sich somit eine geringe Luftkriegsbedrohung erheblich auf das weitere Schicksal psychisch kranker Menschen aus, wie in dieser Untersuchung dargestellt werden konnte. Die reichsweite Verlegungswelle wertete Schmuhl als „Wechselspiel“ zwischen „Druck“ ausgehend von Überbelegungen in den Anstalten, mitunter aufgrund erheblicher Fremdbelegung, und „Sog, der durch die intensivierte Euthanasieaktion“ entstand [29]. Dieser Mechanismus stellt somit einen Erklärungsansatz für diesen Antagonismus dar.

Auch die Provinzialanstalt Münster zeigt in den Verlegungen 1943 Besonderheiten auf. Obwohl aufgrund der Luftkriegsgefährdung der Stadt Münster etwa die Hälfte der stationären Patientinnen und Patienten verlegt wurde, waren lediglich drei Bochumer Opfer betroffen. Als kleinere westfälische Anstalt (1110 im Jahr 1941) könnte eine geringere initiale Anzahl Bochumer Patientinnen und Patienten in der Provinzialanstalt Münster eine plausible Begründung bieten [32]. Schließlich ist die initiale Belegung durch Bochumer Patientinnen und Patienten in den westfälischen Provinzialanstalten nicht erforscht. Um hier dargestellte Verlegungsrouten Bochumer Opfer zu kontextualisieren, stellt dies einen vielversprechenden zukünftigen Forschungsansatz dar.

Die Untersuchung der Sterbezeitpunkte und -orte zeigte Übereinstimmungen im westfälischen Vergleich. So zeigte Walter, dass 1941 46 % der „T4“-Verlegungspatienten durch Kohlenmonoxid in Hadamar getötet wurden [38]. Der Höchstwert von 44 Bochumer Todesopfern in Hadamar im Jahr 1941 bildet diese Entwicklung charakteristisch ab. Nach dem offiziellen „Euthanasie“-Stopp zeigte sich vorerst ein Rückgang der Todesfälle (33) und Bochumer Patientinnen und Patienten starben insbesondere in den „Zwischenanstalten“, in denen sie nach August 1941 verblieben waren. Wie oft dargestellt, führten katastrophale Versorgungszustände zum Anstieg der Patientensterblichkeit in Anstalten [38]. Die Vielzahl von Bochumer Todesopfern in den „Zwischenanstalten“ Weilmünster und Eichberg zeigt eine besondere Akkumulation im westfälischen Vergleich. Insbesondere Weilmünster, als größte aller „Zwischenanstalten“, führte drastische Lebensmittelrationierungen durch und wies zum Ende des Jahres 1943 eine Sterbequote von 55 % auf [38].

Ab 1943 nahm die Anzahl Bochumer Todesopfer rapide zu und es zeigte sich im Rahmen der Verlegungen der „Aktion Brandt“ eine Streuung der Sterbeorte auch in süddeutsche und polnische Gebiete. Diese Entwicklung spiegelt das Resultat der dezentralisiert fortgeführten „Euthanasie“ durch Ärzte und Pflegepersonal wider. Der Höchstwert der getöteten Bochumer Patientinnen und Patienten im Jahr 1944 (93) bildet diese „dezentralisierte Euthanasie“-Periode charakteristisch ab. Die Zunahme der Todesfälle psychisch kranker Bochumer Patientinnen und Patienten in süddeutschen Anstalten (Kaufbeuren, Erlangen, Eglfing-Haar, Regensburg) kennzeichnet das folgenreiche Zusammenspiel von Nahrungsmittelentzug, Überschreiten der Unterbringungskapazitäten und defizitärer Versorgung, wie unter anderem Walter darlegte [38].

Die Untersuchung der Patientencharakteristika ergab einige Besonderheiten. Entgegen bisheriger Forschungsarbeiten wie von Brand-Claussen und Bernet, die von einem eher männlichen „Überlebensvorteil“ berichteten, überwog in den „Euthanasie“-Verlegungen des Bochumer Patientenkollektivs der Männeranteil [5]. Hingegen zeigte auch Hohendorf in einer Stichprobenuntersuchung von „T4“-Opfern einen größeren Anteil weiblicher Opfer [16]. Wenngleich nicht eindeutig nachzuweisen, sind im methodischen Vorgehen ein geringerer Stichprobenumfang und die hohe Dunkelziffer der „Euthanasie“-Opfer, insbesondere der weiblichen Opfer bei ausgeprägter Mortalität der Sterilisationsoperationen bei Frauen, mögliche Erklärungsansätze für diese Diskrepanz [4].

Des Weiteren wiesen die Bochumer Patientinnen und Patienten ein höheres Lebensalter (Männer 43 Jahre, Frauen 44 Jahre) auf als im westfälischen Vergleich [38]. Die geringe Überlebensdauer und das durchschnittliche Sterbealter von 45 Jahren verdeutlichen eindrücklich die Folgen eugenischer und kriegspolitischer Mechanismen im psychiatrischen Versorgungssystem unter nationalsozialistischer Herrschaft.

Die Untersuchung der Diagnosen zeigte sich im westfälischen Vergleich konvergent [13, 38]. Durch die gezielte Tötung psychisch Kranker in den „Tötungsanstalten“, dramatische Versorgungszustände und massive Überbelegung ist die Verschleierung der „Euthanasie“ durch die Deklaration und Pseudodokumentation natürlicher Todesursachen evident und die Ausstellung fiktiver Sterbegründe vielfach belegt [9, 10, 36, 37]. Unter kritischer Beurteilung der dokumentierten Todesarten ist der große Anteil natürlicher Todesursachen vor dem Hintergrund einer dezentralisiert fortgeführten „Euthanasie“ in den Heilanstalten nachvollziehbar. Hierbei fällt insbesondere der hohe Anteil pulmonaler Todesursachen auf, ein Tatbestand, der sich durch die Kombination von durch Vernachlässigung und Nahrungsmittelentzug geschwächten Patienten mit Verabreichung einer Medikation mit Phenobarbital oder Trional (sog. „Luminal-Schema“ nach dem Psychiater und medizinischen Leiter der T4-Aktion H.P. Nitsche) in Zusammenhang bringen lässt [3, 20]. Dieser Verdacht wird auch durch den hohen Anteil an Fällen von „Marasmus“, „Kachexie“ und „Verfall durch Geisteskrankheit“ erhärtet.

Bei der Interpretation der erhobenen Daten zu den Zwangssterilisationen ist zunächst zu beachten, dass nicht alle der in den Erbgesundheitsakten des Stadtarchivs Bochum dokumentierten Zwangssterilisationen ausgewertet wurden, sondern nur die Fälle, die zugleich Teil der Gruppe der vorher ermittelten verlegten Bochumer Patientinnen und Patienten sind. Ob die hier vorgelegten Ergebnisse für die insgesamt im Gesundheitsamt Bochum dokumentierten Fälle repräsentativ sind, ist unbekannt. Wurden etwa zu gleichen Teilen Männer und Frauen sterilisiert, waren, wie Bock darstellte, etwa 90 % der postoperativen Todesopfer weiblich [4]. Aufgrund des methodischen Vorgehens ist insbesondere die Anzahl sterilisierter Bochumer Patientinnen daher vermutlich unterschätzt. Wenngleich nur in 40 Fällen dokumentiert, ist allein die sterilisationsprozessuale Erfassung ein Indiz für die flächendeckende Durchführung von Zwangssterilisationen, wie u. a. Walter zeigte [15, 18]. Auch die zeitliche Entwicklung der Sterilisationsverfahren der Bochumer Opfer zeigte Übereinstimmungen mit den bisherigen Forschungsergebnissen. Das Diagnosenprofil (1. „Schizophrenie“, 2. „angeborener Schwachsinn“, 3. „Epilepsie“) der Sterilisationsopfer glich Walters Untersuchungen über westfälische Sterilisationsopfer in stationärer Unterbringung [38]. In Bochum wurden vergleichsweise viele Zwangssterilisationen durchgeführt. Daher lag es nahe zu schauen, ob und wie viele zwangssterilisierte Patienten aus Bochum stationär in den Provinzialanstalten aufgenommen und später getötet wurden. Auch dieser Zusammenhang konnte hier aufgrund der Datenlage nicht in absoluten Zahlen exakt überprüft werden.

Limitiert wird diese Untersuchung schließlich durch die lückenhafte Überlieferungslage und den methodischen Fokus auf hessische und westfälische Archive, wodurch die gewonnenen Erkenntnisse nur als eine Annäherung an die tatsächlichen Opferzahlen zu verstehen sind. Die Konzentration auf die Bochumer Bürger, von denen jedoch nicht alle sicher und ausreichend vollständig ausgewertet werden konnten, relativiert die Gesamtergebnisse unserer Untersuchung. Die Analyse der Lebenswege psychisch kranker Bochumer Bürger im Nationalsozialismus eröffnet aber exemplarisch Einblicke in die Euthanasie- und Sterilisationsverbrechen und in die damit verbundenen Prozesse der damaligen Zeit.

## Fazit für die Praxis

- Insgesamt wurden 366 psychisch kranke Bochumer Patientinnen und Patienten in den Jahren 1940–1943 im Rahmen der „Euthanasie“-Transporte verlegt. In der „Aktion T4“ stammte der Großteil verlegter Bochumer Patienten aus der Provinzialanstalt Warstein; in den späteren Verlegungen der „Aktion Brandt“ wurde hingegen die Mehrheit aus der Provinzialanstalt Eickelborn nach Süddeutschland verlegt.
- Die „Tötungsanstalt“ Hadamar forderte insgesamt 85 Bochumer Todesopfer. Auch im hessischen Weilmünster, in süddeutschen Anstalten und der heute in Polen befindlichen „Tötungsanstalt“ Meseritz starb ein Großteil der Betroffenen.
- Es überwog im Bochumer Patientenkollektiv der Anteil der Männer (205 Männer, 161 Frauen). Das durchschnittliche Verlegungsalter lag bei 43 bis 44 Jahren, zum Zeitpunkt ihres Todes waren die Opfer durchschnittlich 45 Jahre alt. Es wurden vorwiegend natürliche Todesursachen in den Akten angegeben (20 % kardiovaskulär, 18 % Tuberkulose, 12 % pulmonale Erkrankung).
- Die drei häufigsten Diagnosen des untersuchten Patientenkollektivs waren an 1. Stelle „Schizophrenie“ (44 %), gefolgt von „angeborenem Schwachsinn“ (24 %) und „Epilepsie“ (16 %).
- Von insgesamt 49 in Sterilisationsprozessen verfolgten Bochumer Opfern ist die Durchführung der Zwangssterilisation in 40 Fällen (27 Männer, 13 Frauen) dokumentiert.

## Data Availability

Alle Daten der Studien können auf Anforderung erhalten werden.
